# Effects of various freezing containers for vitrification freezing on mouse oogenesis

**DOI:** 10.1186/s40781-016-0094-4

**Published:** 2016-03-20

**Authors:** Ji Chul Kim, Jae Myeoung Kim, Byoung Boo Seo

**Affiliations:** Department of Animal Resources, Daegu University, Gyeongbuk, 38453 Korea; ROSA Infertility Clinics, Daegu, 41238 Korea; Saewha Hospital, Busan, 47822 Korea; Institute of Life and Environment, Daegu University, Gyeongbuk, Korea

**Keywords:** Mouse embryos, Vitrification freezing methods, EM-grid, OPS, Cryo-loop

## Abstract

**Background:**

In the present study, various freezing containers were tested for mouse embryos of respective developmental stages; embryos were vitrified and then their survival rate and developmental rate were monitored. Mouse two cell, 8 cell, and blastula stage embryos underwent vitrification freezing-thawing and then their recovery rate, survival rate, development rate, and hatching rate were investigated.

**Methods:**

EM-grid, OPS, and cryo-loop were utilized for vitrification freezing-thawing of mouse embryos.

**Results:**

It was found that recovery rate and survival rate were higher in the group of cryo-loop compared to those of EM-grid (*p* < 0.05). Embryonic development rate, two cell embryos to blastocyst, as well as hatching rate were higher in the control group compared to the EM-grid group and OPS group (*p* < 0.05), yet no difference was noted between the control group and cryo-loop group. Development rate and hatching rate of eight cell morulae and blastocysts were all lower in the treatment groups than the control group whilst hatching rate of blastocysts was higher in the control group compared to the groups of EM-grid and OPS (*p* < 0.05); although the cryo-loop group was shown to be slightly higher than other groups, it was not statistically significant.

**Conclusions:**

In the study, we investigate effects of freezing containers on vitrified embryos of respective developmental stages; it was demonstrated that higher developmental rate was shown in more progressed (or developed) embryos with more blastomeres. There was however, no difference in embryonic development rate was shown amongst containers. Taken together, further additional studies are warranted with regards to 1) manipulation techniques of embryos for various vitrification freezing containers and 2) preventive measures against contamination via liquid nitrogen.

## Background

Frozen embryos have been widely utilized for artificial reproductive technologies in both human and animals hence various cryopreservation methods have been proposed. So far, methods for cryopreservation of mouse embryos, bovine blastocysts, and human 2-cell, 8-cell embryos are being well established, validated, and widely adopted [7, 19, 24, 29]. Generally speaking, survival rate varies per species of animals, developmental stages of embryos, and their quality; eggs, in vitro isolated embryos, and damaged embryos (via micro-manipulation, for instance) are known to be more sensitive compared to embryos, in vivo isolated and intact embryos, respectively [22, 25]. Depending upon changes in temperature, these embryos are subjected to damages thus there are considerable difficulties in implementation of cryopreservation. In addition, other experimental conditions including cryopreservation methods, types of cryoprotectant agents, exposure time, and cryopreservation containers, may impact on clinical outcomes. In particular, optimization of cryopreservation methods is required for respective developmental stages given that physical cryopreservation conditions (e.g., permeability of solutes, water contents, freezing rate, and intracellular formation of ice crystals) can consequence different results, potentially through changes in cellular structures and membranous permeability per embryonic development stages and cell cycles [1, 26]. It was demonstrated that survival rate might be elevated in embryos with more blastomeres; further, higher survival rate could be achieved in cryopreservation by utilizing blastocysts compared those of pronuclear embryos or early embryos because of numbers of balstocysts and their sizes. Therefore, better implantation rate could be obtained by virtue of blastocysts that are more suitable for intrauterine environment. Two-cell embryos from mice are known to represent better implantation rate in slow freezing yet it is not the case for cow and sheep embryos. In human, vitrification freezing is a more efficient method and survival rate was found to be relatively better, meaning higher, when vitrified with varying magnitudes per animal species [6, 18]. To note, since blastocysts with large blastocoel cavity are subjected to damage by toxicity of cryoprotectant agents, even before penetration into cells, slow equilibrium at low concentration followed by gradual increment, also known as two-step freezing method, might be more effective. In other studies, improvement of cryoprotectant agents was successful to result better survival rate of embryos regardless of their development stages albeit late blastocysts were more vulnerable to cellular damages, compared to early blastula stage and survival rate of blastocysts was influenced by their developmental stages. In addition to the freezing speed, cryopreservation containers are also one of the important factors influencing mass of cryoprotectant agents and embryos in vitrification freezing; multiple studies have been done using various containers in different developmental stages but they have adopted containers without consideration of embryonic developmental stages. Further, proficiency of researchers should be also taken into account.

Therefore, in order to establish an effective cryopreservation method, we adopted EM-grid, OPS, and cryo-loop, as containers, for cryopreservation of mouse 2-cell, 8-cell, and blastocysts and then, their survival rate, development rate were monitored; furthermore, mitochondrial damages of embryos in respective developmental stages by vitrification freezing were investigated via Mito-Tracker Red staining.

## Methods

### Preparation of animals

Five weeks old, the first generation (F1) female hybrid B6CBAF1 mice (C57BL/CBA) were mated with 10 ~ 15 weeks old male mice of same species that were verified with their reproductive capacity; animals were housed in a cycle of 14 h light and 10 h dark, 40 ~ 60 % humidity, and at 22 ~ 25 °C. Foods and water were provided ad libitum. Female mice were injected with pregnant mare’s serum gonadotropin (PMSG; Sigma-Aldrich, St. Louis, MO, USA) and human chorionic gonadotropin (hCG; Sigma-Aldrich) through intraperitoneal injection with a 48-h interval in order to induce super ovulation. Upon the injection of hCG, female mice were allowed to mate with male and then only female mice with vaginal plugs were utilized. Mice were slaughtered by cervical dislocation after 48, 96, and 144 h from the injection of hCG; 2-cell, 8-cell, and blastocysts were harvested from ampulla tubae uterinae, uterine tube, and uterus using a disposable syringe with 19G needle after perfusion of uterine tube and uterus with phosphate-buffered saline (PBS) supplemented with bovine serum albumin (4 mg/mL). Harvested 2-cell embryos were washed three times using culture medium and cultured in G1 culture medium (G1-5; Vitrolife, Göteborg, Sweden). After 48 h of incubation, culture medium was switched with G2 (G2-5; Vitrolife) while 8-cell embryos and blastocysts were directly washed and cultured in G2 culture medium. For the culture, embryos were maintained at a level of five embryos per 10 uL of mineral oil (Sage, Trumbull, CT, USA) via conventional drop culture method.

### Vitrification freezing and thawing

For vitrification solution, PBS supplemented with 20 % serum substitude supplement (SSS; Irvine Scientific, Santa Ana, CA, USA) was supplemented either 7.5 % ethylene glycol(EG, E-9129, Sigma Aldrich) + 7.5 % dimethyl sulfoxide (DMSO, D-2650; Sigma Aldrich) or 15.0 % EG + 15.0 % DMSO + 0.5 M sucrose. When it comes to the thawing solution, the basic solution (i.e., PBS supplemented with 20 % SSS) was added with either 0.5 M sucrose or 1.0 M sucrose prior to use. Embryos were firstly immersed in the solution supplemented with 7.5 % EG + 7.5 % DMSO for 10 min and then pretreated embryos were moved into the solution of 15.0 % EG, 15.0 % DMSO, and 0.5 M sucrose. Immediately after this, they were loaded on cryopreservation containers and then soaked in liquid nitrogen as soon as possible. Embryos stored in liquid nitrogen more than 2 weeks were retrieved, immersed in 1.0 M sucrose solution at 37 °C followed by 3 min immersion in 0.5 M sucrose solution at room temperature; embryos were then further immersed in the basic solution for 5 min at room temperature followed by additional 5 min incubation at 37 °C. Completely thawed embryos were incubated in an incubator equilibrated at 37 °C and 5 % CO_2_ over 16 ~ 18 h after subsequent washing with G1 and G2 culture medium.

### Vitrification freezing using EM-grid

Embryos to be vitrified were transferred into the last vitrification solution and 1 ~ 3 embryos were loaded on the electron microsope grid (EM-grid; Gilder Co., West Chester, PA, USA). Extra cryoprotectant agents were removed by placing sterilized filter paper underneath the EM-grid. The EM-grid, loaded with embryos, was handled at 37 °C till immerse it into liquid nitrogen and stored in liquid nitrogen within 30 s since exposed to the solution of 15.0 % EG, 15.0 % DMSO, and 0.5 M sucrose.

### Vitrification freezing using OPS

In order to make an Open Pulled Straw (OPS), both sides of 0.25 mL plastic straw were holding and then placed 5 ~ 7 cm above an alcohol lamp for 2 ~ 5 s. And then, the both sides were pulled until the diameter and thickness of middle wall become narrow approximately 0.8 ~ 1.7 and 0.07 ~ 0.15 mm, respectively. The thinnest part of the straw was cut on the bias using a sharp mass. The straw was placed in the 1 uL of vitrification solution so that 2 ~ 3 cm long, cylindrical shape of solution with embryos is transferred through capillary force; immediately after the transfer, the OPS was immersed in liquid nitrogen. To thaw embryos, the OPS was thawed in the thawing solution and then embryos were recovered by blocking the other side of OPS with a finger thereby inducing expansion of inner mass thereof.

### Vitrification freezing using cryo-loop

The cryo-loop has a structure of micro nylon loop (20 um in width and 0.5 ∼ 0.7 mm in diameter) mounted on stainless steel pipe of a cryovial cap (MicroTube^TM^, Hampton research, Aliso Viejo, CA, USA); embryos were loaded on a thin membrane of vitrification solution (less than 1 uL), mounted on a loop of cryovial, and then immersed in liquid nitrogen. All procedures were done in no more than 30 s.

### Staining of mitochondria of mouse embryos

In order to monitor mitochondrial damages on embryos by vitrification, cells were stained using the Mito-Tracker Red (580, M-22425; Molecular Probes, Invitrogen, Waltham, MA, USA). Embryos were immersed in the formaldehyde solution for 20 min to fix mitochondria followed by washing 2 ~ 3 times with PBS added with 0.1 % polyvinyl alcohol (v/v). To stain mitochondria, embryos were exposed to the 1 uM of Mito-Tracker Red solution in an incubator maintaining 37 °C and 5 % CO_2_ for 30 min. Upon completion of staining, embryos were mounted on a slide glass and laser scanned for each sample utilizing the LSM-5 EXCITER program of confocal microscope (at × 400 magnification; ZEISS, Oberkochen, Germany).

### Statistical analysis

The data were statistically analyzed by chi-square test with SPSS program for its significance. *p* value of less than 0.05 was considered not significant.

## Results

### Effects of cryopreservation containers on recovery and survival rate in vitrification of embryos

Mouse embryos underwent vitrification freezing-thawing using respective cryopreservation containers (i.e., EM-grid, OPS, and cryo-loop) and then recovery rate as well as survival rate were investigated (Table 1). After vitrification freezing-thawing, the recovery rates of embryos were 95.5, 89.1, and 92.8 % in the cryo-loop group, the EM-grid group, and the OPS group, respectively; the cryo-loop group represented significantly higher recovery rate compared to the EM-grid group (*p* < 0.05). On the other hand, the survival rate were 92.5, 83.6, and 82.4 % in the group of cryo-loop, EM-grid, and OPS, respectively which is similar to the recovery rate. There was a significant difference between the cryo-loop and others (*p* < 0.05).

**Table 1:** Recoverable and survival rate of vitrified embryos according to various vitrification containers

Method	No. of embryos	No. (%) of embryo	
		Recovered	Survived
EM-grid	128	114 (89.1)^b^	107 (83.6)^b^
OPS	125	116 (92.8)^ab^	103 (82.4)^b^
Cryo-loop	134	128 (95.5)^a^	124 (92.5)^a^

^a,b^ With the same columns, values with different superscripts differ significantly (*p* < 0.05)

### Effects of cryopreservation containers on development of embryos in different developmental stages in vitrification freezing

Development rate and hatching rate of mouse 2-cell embryos were investigated after vitrification freezing-thawing utilizing EM-grid, OPS, and cryo-loop, as cryopreservation containers (Table 2). The development rate of 2-cell embryos to 8-cell embryos was 89.6 % in the control group which was not statistically different compared to those of the groups of EM-grid (76.1 %), cryo-loop (85.4 %); to note, however the OPS group exhibited significantly lower development rate (74.4 %; *p* < 0.05). The development rate and hatching rate of 2-cell embryos (to blastocysts and hatched blastocysts) were 83.3 % and 79.2 % respectively in the control group. There were significant differences between the control group against the EM-grid group (60.9 and 54.3 %) as well as the OPS group (60.5 and 58.1 %) yet no difference was noted in the cryo-loop group (70.8 and 64.6 %; Table 2). Albeit no significant difference was found between developmental stages of embryos amongst treatment groups, the cryo-loop group had higher development rate and hatching rate in respective developmental stages (85.4, 70.8, and 64.6 % for 8-cell embryos, blastocysts, and hatched blastocysts, respectively) than those of the EM-grid group (76.1, 60.9, and 54.3 %) as well as the OPS group (74.4, 60.5, and 58.1 %).

**Table 2:** Subsequent embryonic developmental rate of vitrified 2-cell embryos according to variousvitrification containers

Method	No. of 2 cells	No. (%) of embryo developed to		
		≥8 cell	BL*	Hatched BL*
Control	48	43 (89.6)^a^	40 (83.3)^a^	38 (79.2)^a^
EM-grid	46	35 (76.1)^ab^	28 (60.9)^b^	25 (54.3)^b^
OPS	43	32 (74.4)^b^	26 (60.5)^b^	25 (58.1)^b^
Cryo-loop	48	41 (85.4)^ab^	34 (70.8)^ab^	31 (64.6)^ab^
Total (Mean)^†^	137	108 (78.8)	88 (64.2)	81 (59.1)

^a, b^With the same columns, values with different superscripts differ significantly (*p* < 0.05)

*blastocyst

^†^EM-grid + OPS + Cryo-loop - Control

In mouse 8-cell embryos, development rate and hatching rate were monitored after vitrification freezing-thawing utilizing EM-grid, OPS, and cryo-loop, as cryopreservation containers (Table 3). The development rate and hatching rate of 8-cell embryos to morulae embryos/blasocysts and hatched blastocysts in the control group were higher than all other treatment groups (95.2, 92.9, and 90.5 % for morulae embryos, blastocysts, and hatched blastocysts, respectively; *p* < 0.05) whilst the cryo-loop group (81.0, 78.6, and 73.8 %) had better developmental rate and hatching rate compared to the groups of EM-grid (70.0, 70.0, and 62.5 %) and OPS (73.2, 73.2, and 68.3 %) yet no statistical significance was found.

**Table 3:** Subsequent embryonic developmental rate of vitrified 8-cell embryos according to variousvitrification containers

Method	No. of 8 cells	No. (%) of embryo developed to		
		Morulae	BL*	Hatched BL*
Control	42	40 (95.2)^a^	39 (92.9)^a^	38 (90.5)^a^
EM-grid	40	28 (70.0)^b^	28 (70.0)^b^	25 (62.5)^b^
OPS	41	30 (73.2)^b^	30 (73.2)^b^	28 (68.3)^b^
Cryo-loop	42	34 (81.0)^b^	33 (78.6)^b^	31 (73.8)^b^
Total (Mean)^†^	123	92 (74.8)	91 (74.0)	84 (68.3)

^a, b^With the same columns, values with different superscripts differ significantly (*p* < 0.05)

*blastocyst

^†^EM-grid + OPS + Cryo-loop - Control

### Effects of cryopreservation containers on hatching rate of vitrified blastocysts after vitrification freezing-thawing

**Fig. 1:**
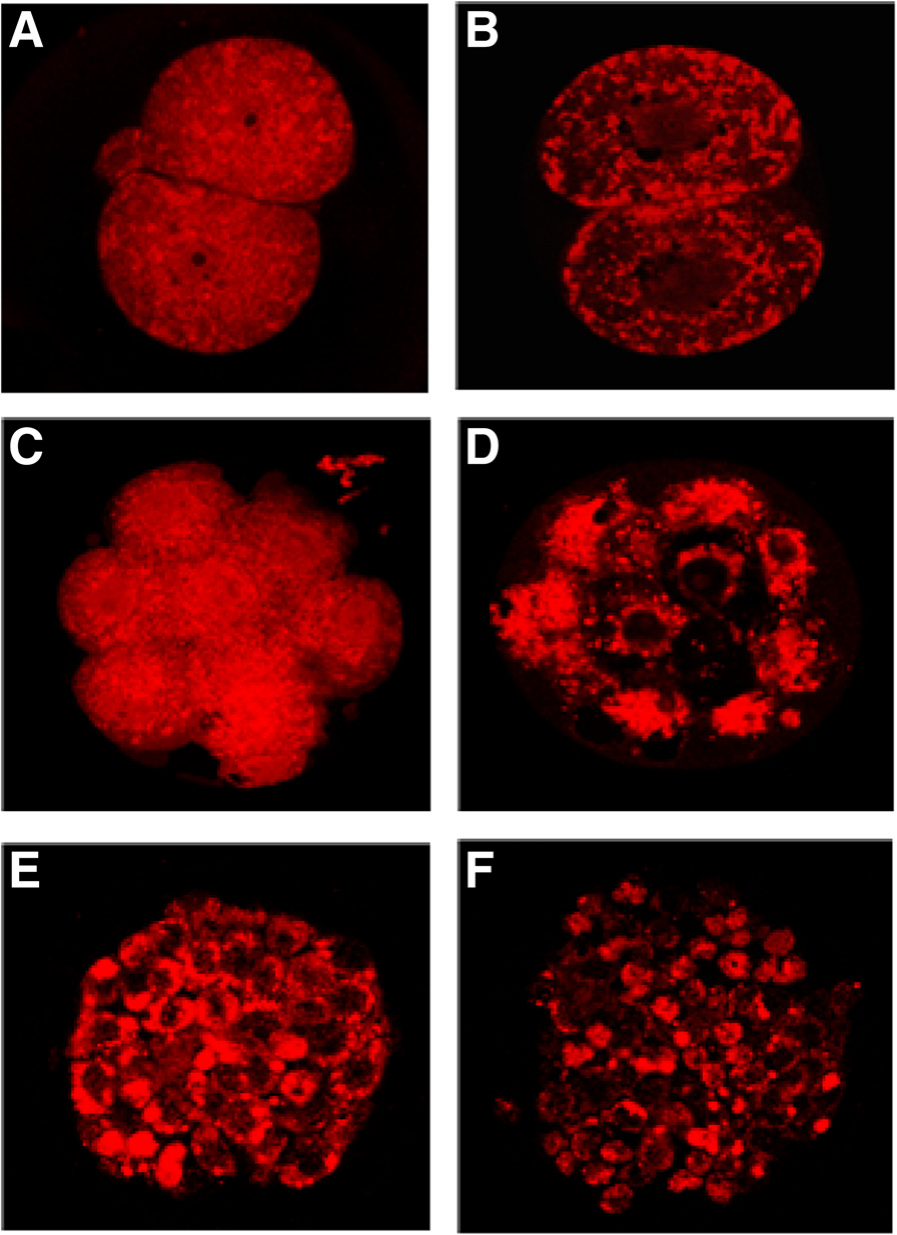
Laser-scanning confocal microscopy image of mitochondria stained by Mito-Tracker at each developmental stage of mouse embryos according to in vitro culture and vitrification (×400). Red : mitochondria stained by Mito-Tracker. **a c** and **e** normal 2-, 8-cell embryo and blastocyst. **b**, **d** and **f** cryo-shocked 2-, 8-cell embryo and blastocyst

The hatching rate of mouse vitrified blastocysts and effects of cryopreservation containers (i.e., EM-grid, OPS, and cryo-loop) were summarized in the Table 4. The hatching rate of mouse blastocyst was found to be 95.2 % in the control group which was significantly higher than the EM-grid group (83.3 %) and the OPS group (78.0 %; *p* < 0.05). The cryo-loop group had slighter hatching rate than other treatment groups yet was on the border line of statistical significance (*p >* 0.05). In addition mouse embryos were stained using the Mito-Tracker Red to monitor mitochondrial damages, possibly caused by vitrification freezing; 2-cell, 8-cell embryos and blastocysts were stained and photographs of unfrozen/frozen embryos were compared (Fig. 1).

**Table 4:** Hatching rate of vitrified blastocysts according to various vitrification containers

Method	No. of blastocysts	No. (%) of embryo developed to hatched BL*
Control	42	40 (95.2)^a^
EM-grid	42	35 (83.3)^b^
OPS	41	32 (78.0)^b^
Cryo-loop	44	38 (86.4)^ab^
Total (Mean)^†^	127	105 (82.7)

^a,b^With the same columns, values with different superscripts differ significantly (*p* < .05)

*blastocyst

^†^ EM-grid + OPS + Cryo-loop - Control

## Discussion

The EM-grid represents high thermal conductivity and enables to quickly lower temperature since only small volume of cryoprotectant solution is immersed in liquid nitrogen; further, it is easy to store samples using an assembly, consisting of cryovial cap and goblet [24]. Kim and Lee [10] however, reported that their recovery rate of embryos could be poor compared to other cryopreservation containers because of the direct exposure (of embryos on the grid) to exterior. This is in a good agreement with results herein. In contrast, the OPS is characterized with relatively faster freezing time, compared to other conventional straw methods but requires small loading volume with quick filling by virtue of thin inner wall of pulled straws thereby improving vitrification as well as clinical outcomes [28]. Lastly, when it comes to the cryo-loop, less than 1 uL of vitrification freezing solution is loaded on a thin membrane followed by immersion into liquid nitrogen and closure which makes samples to be easily stored in cryovials and labeled [20]. Although vitrification freezing methods utilizing these containers (e.g., EM-grid, OPS, and cryo-loop) represent fast freezing rate due to small amount of vitrification solution required as well as direct immersion into liquid nitrogen, highly skillful techniques and contamination risks from liquid nitrogen (e.g., virus contamination) might be expected [13].

Kasai et al. [7] demonstrated 98 and 51 % of survival rate and pregnancy rate, respectively in which mouse morulae embryos were vitrified and thawed using a straw; in the present study, we found the similar survival rate of mouse embryos by using the cryo-loop (92.5 %). And Kim et al. [9]. demonstrated 100.0 and 96.2 % of recovery rate and survival rate of human embryos underwent vitrification freezing using an OPS; the percentage for partial damages on blastomeres was shown to be 19.0 % which is considerably lower than other methods but results herein demonstrated slightly lower recovery rate and survival rate (92.8 and 82.4 %, respectively) in the OPS group. Lee et al. [16]. vitrified and thawed mouse blastocysts using EFS40 and VS solution in parallel with the EM-grid; in this, the recovery rates and survival rates were shown to be 73.7 and 66.5, and 80.5 and 66.0 %, respectively. In contrast, we found slightly higher recovery rate and survival rate (89.1 and 83.6 %) in the EM-grid group (Table 1). When bovine embryos were vitrified and thawed using the EM-grid, OPS, and cryo-loop, no difference was noted in their survival ratewhich is not similar to our observation; in the study, the cryo-loop group had significantly higher survival rate compared to those of EM-grid and OPS [17].

Previously, it was reported that higher survival rate of embryos could be achieved in which 1) further developed blastocysts with increased blastomeres, were utilized, 2) cryopreservation was performed as quickly as possible^,^, and 3) mouse blastocysts were vitrified [2, 12, 27, 32]. Similarly, we found that hatching rate was higher for further developed embryos in an order of 2-cell, 8-cell, and blastocysts. Lee et al. [17] vitrified in vitro bovine 8-cell, morulae embryos, and blastocysts using the EM-grid, OPS, and cryo-loop in order to monitor their hatching rates; in results, no difference was shown between groups. In like manner, we found that vitrification containers (i.e., EM-grid, OPS, and cryo-loop) were not significantly impacting on hatching rate for vitrified embryos regardless of their developmental stages except for the control group. On the other hand, Lee et al. [16], also reported that the hatching rates for 8-cell embryos, morulae embryos, and blastocysts were 32.0, 50.1 and 69.8 %, respectively in each developmental stage, demonstrating further developed embryos tend to represent higher hatching rate. Under our experimental conditions, the hatching rates for 2-cell, 8-cell embryos, and blastocysts were 59.1, 68.3 and 82.7 %, respectively that is in agreement previous investigations including the study of Bolton et al. [2].

In addition to freezing rate, selection of cryopreservation containers has been known as one of the important factors, influencing outcomes of vitrification given that it could reduce volume of cryoprotectant agents and embryos; depending upon developmental stages, OPS, cryotop, and microdrop [3, 8, 14, 23] were used for egg and early embryos while plastic straw, EM-grid, and cryo-loop have been utilized for blastocysts [5, 21, 30, 31].In recent, various cryopreservation containers are being chosen without consideration regarding developmental stages of embryos, and clinical outcomes might vary per researchers’ degree of skill in vitrification. However, in the present study, we were not able to find significant differences in their hatching rate as well as development rate in response to the selection of cryopreservation containers.

Kim et al. [11] reported that hatching rates of bovine 2-cell, 8-cell, morulae embryos and blastocysts were lower than the control group when a OPS was utilized for vitrification freezing while in our experiment, the OPS group had lower hatching rates for 2-cell, 8-cell embryos, and blastocysts. Park et al. [24]. reported favorable outcomes of vitrification freezing for bovine blastocysts, vitrified using the EM-grid with EFS solution. In a similar manner, Cho et al. [4] have demonstrated good results for human blastocysts when vitrification freezing was performed using the EM-grid. In the study, satisfactory hatching rates were achieved without showing significant difference between blastocysts of the EM-grid group and other treatment groups. Lane and Gardner [15] reported that there is no difference in developmental rate, hatching rate, and implantation rate of mouse embryos compared to the control group when a cryo-loop was adopted whereas the hatching rate of 8-cell embryos (to blastocysts) was significantly different in the cryo-loop group compared to the control group even though no effect was shown in the 2-cell embryos and blastocysts of cryo-loop groups. In addition, Lee et al. (2006) obtained blastocysts from 72-h in vitro culture of mouse 2-cell embryos and tested effects of various cryopreservation containers on hatching rates after vitrification freezing. The hatching rates of embryos were 60.9, 40, and 65 % for the EM-grid, OPS, and cryo-loop, respectively. As results, the OPS group had lower hatching rate compared to the other two yet such trend was not shown in the study.

Taken altogether, we investigated effects of cryopreservation containers on embryos in different developmental stages and found that development rates of embryos were higher when further developed embryos were adopted with increased blastomeres in vitrification freezing although selection of cryopreservation containers did not impact on their developmental rate. In future, additional studies are warranted with regards to development of cryopreservation containers, manipulation techniques for vitrification, and preventive measures against contamination through liquid nitrogen.

## Conclusions

In the study, we investigate effects of freezing containers on vitrified embryos of respective developmental stages; it was demonstrated that higher developmental rate was shown in more progressed (or developed) embryos with more blastomeres. There was however, no difference in embryonic development rate was shown amongst containers. Taken together, further additional studies are warranted with regards to 1) manipulation techniques of embryos for various vitrification freezing containers and 2) preventive measures against contamination via liquid nitrogen.
